# Yellow Fever in Woodville, Mississippi

**Published:** 1844-12

**Authors:** 


### YELLOW FEVER IN WOODVILLE, MISSISSIPPI.

During the past autumn, the newspapers announced the occurrence of yellow fever, at Woodville, the seat of justice of Wilkinson county, Mississippi. Anxious to know the reality of this report, we, some time since, addressed a letter to several of its physicians, requesting accurate information; but as yet have received the reply of one only—Dr. C. H. Stone. From the detail of symptoms, in his communication, no reasonable doubt can be entertained that the late epidemic was, in fact, true yellow fever. Four physicians, who had seen veritable cases of that disease, pronounced the black vomit thrown up before death at Woodville, to have all the characteristics of the genuine; and, with one exception, all who had previously experienced that disease in New Orleans and the West Indies, now escaped it; finally, Dr. S., who has practised medicine 13 years in Wilkinson county, never before saw a fever of the same type.

If we admit, as it seems we must, that this was a real invasion of yellow fever, it presents a matter of intense interest, to all who are engaged in the investigation of the cause of that disease. Woodville is situated 12 or 15 miles from the Mississippi river, 'on a ridge, with a dry surface of sand and clay; not filthy, or, at least, not more so than in common years, and 10 or 12 miles from any swamp.' How then can the fever be referred to any of the commonly assigned local causes? On the other hand, the disease was only—scarcely—sporadic in New Orleans, and during the present year, entirely absent from all of the river towns above that city. If introduced at Woodville, whence did it come? For the present Dr. S. has declined giving us a statement of facts relative to its origin, but says, that when presented, they will carry 'conviction to the minds of all, not only that yellow fever had its origin in *Woodville*, but *elsewhere* in Wilkinson county.' Some facts will appear, also, he adds, 'to show that the *common* causes to which this disease is attributed, have no part in its organization, or that it may occur without them.'. We shall await his development with impatience.

